# Cure of Burns

**Published:** 1844-05-18

**Authors:** 


CURE OF BURNS.

Dr. Fenaille proposes the following remedy in the treatment of burns. After opening the vesicles, if they are formed, the part is dipped in cold water and then plunged, still wet, in flour, keeping it there for a minute or two; by this means a certain quantity adheres to the part and prevents the access of the air. It is remarkable that the flour falls in scales from the surrounding parts the next day, whilst on the burn it remains adherent.—*Ibid.*

